# Treatment of B-cell precursor acute lymphoblastic leukemia with the Galectin-1 inhibitor PTX008

**DOI:** 10.1186/s13046-018-0721-7

**Published:** 2018-03-27

**Authors:** Helicia Paz, Eun Ji Joo, Chih-Hsing Chou, Fei Fei, Kevin H. Mayo, Hisham Abdel-Azim, Haike Ghazarian, John Groffen, Nora Heisterkamp

**Affiliations:** 1 Section of Molecular Carcinogenesis, Division of Hematology/Oncology and Bone Marrow Transplantation, The Saban Research Institute of Children’s Hospital Los Angeles, Los Angeles, CA 90027 USA; 2 Department of Systems Biology, Beckman Research Institute City of Hope, Monrovia, CA USA; 3 Department of Biochemistry, Molecular Biology & Biophysics, University of Minnesota, Health Sciences Center, 6-155 Jackson Hall, 321 Church Street, Minneapolis, MN 55455 USA; 4 Division of Hematology/Oncology and Bone Marrow Transplantation, Children’s Hospital Los Angeles, Los Angeles, CA 90027 USA; 5 Department of Surgical Oncology, UCLA, Los Angeles, CA 90095 USA; 6 Division of Immunobiology, Cincinnati Children’s Hospital Medical Center, Cincinnati, OH 45229 USA; 7 Pathology Department, University of Alabama, Birmingham, AL USA

**Keywords:** Lymphoid neoplasia, Co-culture, Stroma, Migration, Adhesion

## Abstract

**Background:**

Drug resistance of B-cell precursor acute lymphoblastic leukemia (BP-ALL) cells is conferred by both intrinsic and extrinsic factors, which could be targeted to promote chemo-sensitization. Our previous studies showed that Galectin-3, a lectin that clusters galactose-modified glycoproteins and that has both an intracellular and extracellular location, protects different subtypes of BP-ALL cells against chemotherapy. Galectin-1 is related to Galectin-3 and its expression was previously reported to be restricted to the MLL subtype of BP-ALL.

**Methods and results:**

Here, we report that Galectin-1 is expressed at different levels in and on different subclasses of BP-ALLs. Bone marrow plasma also contains high levels of Galectin-1. PTX008 is an allosteric inhibitor which inhibits Galectin-1 but not Galectin-3-mediated agglutination. The compound reduces migration of BP-ALL cells to CXCL12 and OP9 stromal cells and inhibits fibronectin-mediated adhesion. It also affects cell cycle progression of BCP-ALL cells. PTX008 is cytostatic for BP-ALL cells even when these are co-cultured with protective stroma, and can sensitize ALL cells to vincristine chemotherapy in vitro and in mice.

**Conclusions:**

PTX008 inhibits multiple functions that contribute to BP-ALL survival. The effects of Galectin-1 inhibition on both BP-ALL cell proliferation and migration suggest both the leukemia cells as well as the microenvironment that protects these cells may be targeted.

**Electronic supplementary material:**

The online version of this article (10.1186/s13046-018-0721-7) contains supplementary material, which is available to authorized users.

## Background

Galectins are carbohydrate-binding proteins defined by their specificity for β-galactose-containing glycoproteins and glycolipids, and which regulate immune cell functions [1]. Galectin-1 and Galectin-3 contain a single carbohydrate recognition domain as well as domains that allow homo-dimerization and oligomerization, respectively [2, 3]. These Galectin domains mediate crosslinking of cell surface glycoprotein receptors, glycolipids on the same cell, between cells and in cell-matrix interactions, ultimately triggering intracellular signaling. Additionally, Galectins also can interact with proteins inside the cell, although such interactions typically are not protein-carbohydrate, but protein-protein mediated through residues outside the carbohydrate recognition domain [3].

Galectins have been extensively implicated in cancer, specifically regulating cell transformation, apoptosis, proliferation, migration, invasion, and angiogenesis [2]. We recently showed that Galectin-3 levels in bone marrow plasma of B-cell precursor acute lymphoblastic leukemia (BP-ALL) samples are elevated compared to controls. Galectin-3 moreover protected BP-ALL cells against chemotherapy-induced apoptosis [4]. Interestingly, we found that bone marrow stromal cells secrete and transfer Galectin-3 protein to BP-ALL cells via exosomes. Such stromal exosomes also contain Galectin-1 [5]. Additionally, high Galectin-1 expression was previously reported using immunohistochemistry in Hodgkin lymphoma samples, and in mixed lineage leukemia-rearranged BP-ALL cells by FACS and Western blot [6, 7]. Galectin-1 may promote survival of hematological malignancies through direct action on tumor cells but also through effects on the tumor microenvironment. Increased endogenous expression of Galectin-1 was observed to protect K562 chronic myelogenous leukemia cells against adriamycin and imatinib, and in a mouse lymphoma model, Galectin-1 inhibited CD20 mAb-dependent, macrophage-mediated cell killing [8, 9]. High Galectin-1 levels measured by gene expression profiling correlated with worse outcome in multiple myeloma, and knockdown of Galectin-1 in multiple myeloma cells resulted in smaller tumor formation and less lytic bone damage in an intra-tibeal injection model [10].

Because the function of Galectin-1 in BP-ALL has not been investigated, we assessed this through the use of PTX008, an allosteric inhibitor of Galectin-1 [11–13]. We found that PTX008 inhibits Galectin-1-regulated cell aggregation, adhesion, migration of BP-ALL cells, and sensitizes the ALL cells to treatment with chemotherapy. These results suggest that PTX008, because it inhibits multiple pro-survival functions of Galectin-1, could be useful in combination with chemotherapy for the treatment of specific BP-ALLs that have high expression of this immunomodulator.

## Methods

### Cells, culture and drugs

**Fig. 1:**
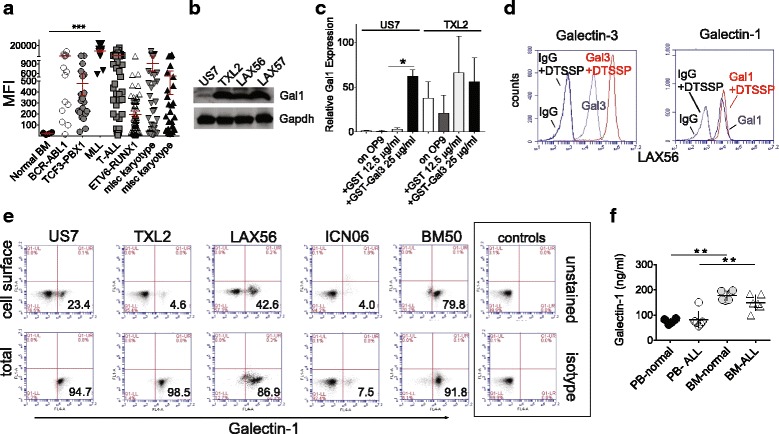
Galectin-1 is expressed in different BP-ALL subtypes. **a** Meta-analysis of GSE28497 representing 270 ALL diagnosis samples and four CD19 + CD10+ control normal B-cell precursors (normal BM). ALLs are subdivided into categories based on karyotype abnormalities as indicated [18] The category MLL includes BP-ALLs with different MLL gene rearrangements (bars, mean ± SEM; ***p≤0.001, 95% CI, 1-way ANOVA; other comparisons, ns.). Each symbol represents one sample. MFI, mean fluorescent intensity. **b** Western blot analysis of Ph-negative US7, Ph-positive TXL2, relapse LAX56 and diagnosis LAX57 BP-ALL patient-derived lines (Genetex Galectin-1 Abs). **c** Real-time RT/PCR for Galectin-1 mRNA on the indicated ALLs cultured for 24 h in complete medium but without OP9 stroma, then stimulated with the indicated recombinant proteins for an additional 24 h. Two samples represent ALL cells grown with irradiated OP9 stroma as indicated. Values shown were normalized to a reference gene, GAPDH, and are comparisons with non-treated US7 cells. One of two experiments, similar results. US7 GST-Gal3 compared to GST *p < 0.05. 95% CI, 1-way ANOVA. **d** FACS analysis for Galectin-1 and Galectin-3 with and without cross-linking with DTSSP to stabilize Galectin-1 and Galectin-3 protein complexes on the cell surface. Antibodies used are indicated above the panels. **e** FACS analysis (antibodies- R&D Systems Galectin-1) for cell surface and total Galectin-1 expression in the indicated ALLs co-cultured on OP9 stromal cells mitotically inactivated by mitomycin C treatment. Percentages indicated are for the lower right-hand quadrant. **f** Galectin-1 levels measured by ELISA consisting of GeneTex anti-Galectin-1 capture antibodies and Abcam anti-Galectin-1 primary detection antibodies with secondary HRP-conjugated goat anti-mouse antibodies. Values represent ng/ml in undiluted control and ALL human plasma samples (*n* = 5–6 for each) from PB and BM collected with anticoagulant after removal of the cellular component

All BP-ALL studies, except where noted, were conducted using a co-culture system in which the leukemia cells are grown with mitotically inactivated murine OP9 stromal cells in αMEM with 20% FBS, 1% L-glutamine and 1% penicillin/streptomycin (Life Technologies, Grand Island, NY). OP9 cells were mitotically inactivated by exposure to ≈ 8000 cGy γ irradiation. In other experiments (Figs. 1e, 5e and 6c, d) OP9 cells were mitotically inactivated by treatment with 10 μg/ml mitomycin C (Sigma cat M4287) for 3 h in complete medium. Selected lots of FBS that supported the co-culture included JR Scientific cat 43,640 lot K051–6; Life Technologies cat 16,140,071 lot 1,642,038 US Qual HI; and Atlanta cat S11550H lots D14047 and L15124. OP9 (CRL-2749) was obtained from the American Type Culture Collection. Patient-derived BP-ALLs include previously described [14–17] TXL2 (diagnosis sample, Ph-positive, p210 BCR-ABL1 wild type), ICN06 (diagnosis; tel-AML1), US7 (no known karyotype abnormalities; bone marrow sample, adult, at diagnosis), LAX57 (diagnosis; 96% blasts; CD45 +/−(dim to negative), CD19 + CD10 + CD20+/− (dim to negative), CD22 + CD34 + SIg- with [t(1;9)(q44;p22)]), and LAX56 (relapse; 89% blasts; CD45+/−(dim to negative), CD19 + CD10 + CD20+/− (dim to negative), CD22 + CD34 + SIg-; with [t(Y;7)(p11.3;p13)]). LAX56 and LAX57 grew directly on OP9 stroma from Ficoll-purified bone marrow mononuclear cells. TXL2, ICN06 and US7 were passaged in NOD.Cg-Prkdcscid IL2rgtm1Wjl/SzJ (NSG) mice (Jackson Labs, Bar Harbor, ME), then grown on OP9 stroma. BM50 was a CD19-CD22+ relapse after bone marrow transplant and CART19 treatment. BM50 cells were passaged in NSG mice as PDX, then grown on OP9 stroma. Human specimen collection protocols were reviewed and approved by Children’s Hospital Los Angeles Institution Review Board (IRB) [Committee on Clinical Investigations] (CCI). Collections were in compliance with ethical practices and IRB approvals. Nilotinib (AMN107), obtained from Novartis (Basel, Switzerland), was dissolved in DMSO and stored at − 20 °C. A vincristine sulfate solution was obtained from Hospira Worldwide Inc. (Lake Forest, IL, USA). PTX008 (− 1 in Fig. 5e) was from Kevin Mayo. PTX008 (− 2 in Fig. 5e) was purchased from Axon Medchem LLC (Reston, VA 20191, cat # 2332) and was also used in Fig. 6c, d and in Additional file 1: Figure S2 panel c.

### Galectin-1 RNA and western blot expression analysis

Gene expression analysis- Meta-analysis was performed for LGALS1 (*Galectin-1)* expression on a data set of RNAs from 270 pediatric bone marrow samples of ALL at diagnosis and 4 normal B-cell progenitor bone marrow samples selected for CD19 and CD10 on HG-U133A Affymetrix arrays [18]. Processed data in the series matrix files from GEO Datasets accession GSE28497 [18] represent values normalized by MAS5.0 with MFI values calculated to a median target intensity of 500. Txt file values for probe set ID 201105_at imported into Excel were manually extracted into Prism5.0. Coustan-Smith et al. reported (Table S2 in [18]) that 78.9% of the leukemia samples overexpressed *LGALS1*, defined as 25% or more of B-lineage ALLs compared to normal precursor B-cells.

Real-time RT/PCR- For RNA analysis, cells were cultured in complete medium for 24 h or plated on irradiated OP9 cells. They were then stimulated with recombinant GST or GST-Gal3 purified with a step to remove endotoxin as described, [5] for 24 h and harvested. RNA was isolated using Trizol. cDNA was generated with random primers and an ABi High Capacity cDNA reverse transcriptase kit according to the supplier’s instructions. Primers used for reverse transcription/real time PCR of human *LGALS1* were: forward 5-CTC TCG GGT GGA GTC TTC TG-3 and reverse: 5-GAA GGC ACT CTC CAG GTT TG-3. Samples were run in triplicate and data were analyzed using comparative Ct, with all samples being normalized to non-treated US7 cells.

Western blotting- Cells were lysed by a 20-min incubation in RIPA buffer (50 mM Tris-HCL, pH 8.0, 150 mM NaCl, 1% Triton X-100, 0.5% deoxycholate, 0.1% SDS) containing 2 μg/mL aprotinin, 10 μg/mL leupeptin, 1 μg/mL pepstatin A, 1 mM PMSF, 10 mM sodium fluoride, 1 mM sodium orthovanadate. Membranes were reacted with antibodies against Galectin-1 (cat 101,566, Genetex, Irvine, CA), pSrc (cat 2101S Cell Signaling Technology [CST] (Boston, MA), p44/42 Erk (cat 4377S CST), Src (cat 2109 CST), Erk (sc-94, Santa Cruz Biotechnology, Dallas, TX), Galectin-3 (cat 125,402, Biolegend, San Diego, CA). Gapdh (cat 627,408, Genetex, Irvine, CA) was used as a loading control.

### Galectin-1 flow cytometry

To determine the levels of Galectin-1 on the surface of ALL cells, all ALL cells in the plate (except where indicated), were collected from OP9 cell co-cultures. ALL cells were washed 1× with FACS buffer (PBS, 2% BSA, 0.1% sodium azide) prior to blocking with human FcR blocking reagent (Miltenyi Biotech, San Diego, CA) according the manufacturer’s instructions. Cells (1 × 10^6^) were then incubated with 2.5 μg Galectin-1 antibody (Fig. 1: cat AF1152 R&D Systems, Minneapolis, MN; Fig. 3: cat 101566 Genetex, Irvine, CA) conjugated to CF647 (Mix-n-stain, Sigma-Aldrich, St. Louis, MO), for 45 min at 4 °C. Control cells were stained with rabbit APC-conjugated IgG. Cells were washed 2× with FACS buffer prior to analysis on a BD Accuri C6 cytometer (BD Biosciences, San Jose, CA). PE-conjugated Galectin-3 antibodies were from Biolegend (cat 126706).

DTSSP (3,3′-dithiobis[sulfosuccinimidylpropionate]; Thermo Scientific, Waltham, MA) was dissolved in PBS. LAX56 cells (6 × 10^6^) were resuspended in PBS and incubated with 2 mM DTSSP for 30 min at room temperature (RT). The reaction was terminated by addition of 20 mM Tris, pH 7.5 and incubation for 15 min at RT. For Galectin displacement assays, GST-Galetin-3 obtained from [19] was generated as previously described. A plasmid encoding bacterially-expressed His-tagged Lgals-1-pMCSG7 (clone HSCD00343182) was obtained from DNASU (Arizona State University). Galectin-1 protein was purified using standard procedures. US7 and LAX56 cells in co-culture with OP9 stromal cells were treated for one hour with DMSO or 10 μM PTX008, followed by further exposure to medium alone or to 50 mM lactose, 20 μM recombinant human Galectin-1 or 20 μM recombinant human Galectin-3 for 24 h. Suspension cells were collected and analyzed by FACS for cell surface Galectin-1 or Galectin-3.

### Galectin-1 ELISA

Human bone marrow and PB were collected in EDTA tubes and the cellular component removed by centrifugation. Plasma samples were diluted 10× and values calculated based on a standard curve as described below. We tested the same samples independently using a commercial human Galectin-1 ELISA (used in Additional file 1: Figure S1) and an ELISA made as follows (used in Fig. 1f). As described above, human recombinant His-tagged and TEV protease-site containing Galectin-1 protein was produced in E-coli Rosetta from pMCSG-7-Gal1 plasmid (DNASU clone HsCD00343182). Recombinant protein was isolated on a Ni-NTA agarose (Thermo-Fisher) column and treated overnight with TEV protease (5 u/ml). TEV protease, undigested His-Galectin-1 protein and His tag were removed by collecting the flow-through of a second Ni-NTA column. Samples were run on SDS-PAA gels to check the concentration and purity of the protein. Galectin-1 was serially diluted from 100 ng/ml to 0.03 ng/ml to generate a standard curve. A 96-well plate was coated with 10 μg/ml capture antibody (GeneTex, GTX101566) overnight at 4 °C. After blocking with 5% BSA in PBST (containing 0.05% Tween-20) at RT for 1 h, plasma samples were diluted in 10 times with PBS and applied into each well in 100 μl for 2 h incubation at RT. After washing, 2.5 μg/ml detection antibody (Abcam, ab58085) was added and the plate was incubated at RT for 2 h. HRP-conjugated goat anti-mouse IgG (H + L) (Invitrogen, 62–6520) as secondary antibody was diluted 1:400, and added to the plate for 1 h RT incubation. To measure absorbance, 100 μl ABTS Peroxidase Substrate (KPL, 50–66-00) was added into each well followed by 30 min incubation. The result was detected by absorbance at 405 nm and sample Galectin-1 levels determined using the standard curve. Human plasma samples from peripheral blood and bone marrow were as described previously [4]. For the results shown in Additional file 1, an ELISA was purchased from R&D Systems.

### Cell agglutination assay

LAX56 cells (2 × 10^4^) were seeded into wells of a 24-well plate and incubated with DMSO or 10 μM PTX008 in the absence of OP9 cells. Cells were then treated with 20 μM Galectin-1 or GST-Galectin-3 or GST recombinant proteins for four hours. Cells were imaged using an Olympus 1X71 inverted microscope with digital camera. Agglutinated cell clusters defined as cell aggregates of 200 μm or larger were counted from three different images of three wells.

### Cell adhesion assay

A 96-well plate was coated overnight at 4 °C with 5 μg/mL fibronectin in PBS. Negative control wells were coated with 0.01% poly-L-lysine (Sigma-Aldrich, St. Louis, MO), for 20 min at RT. All wells were washed with 0.1% BSA in PBS prior to blocking with 2% BSA in PBS for 1 h at RT. After blocking, wells were washed twice with 0.1% BSA in PBS. LAX56 cells were labeled with 5 μM Calcein AM (ThermoFisher Scientific, Waltham, MA) for 30 min at 37 °C. Cells were washed 2× with PBS prior to seeding at 5 × 10^4^ cells/well in αMEM base media +/− PTX008 or DMSO for 30 min. As a positive control for Galectin-1 inhibition, cells were incubated with various concentrations of lactose (Sigma-Aldrich, St. Louis, MO). After 30 min, the plate was read at 485 nM on a Synergy HTX Multi-Mode Reader (BioTek, Winooski, VT). The plate was washed two more times with 0.1% BSA in PBS, prior to reading again at 485 nM. The percent adherent cells was calculated using the following formula: adhesion = [(MFI _post wash_-MFI _background_)/ (MFI _pre wash_-MFI _background_)] × 100.

### Cellular migration

Migration of US7 and LAX56 cells was tested using a Transwell assay (5 μm pore size). US7 and LAX56 cells were washed 1× with base αMEM prior to resuspension in αMEM + 1% FBS and either DMSO vehicle or 10 μM PTX008. 100 μL of media containing 5 × 10^5^ cells was placed in the upper chamber and 600 μL of media with or without 200 ng/mL SDF-1α (PeproTech, Rocky Hill, NJ) in the lower chamber. For migration of leukemia cells towards OP9 cells, a confluent layer of irradiated OP9 cells was plated in the well 24 h prior to performing the Transwell assay. After overnight incubation, upper chambers were removed and cells that had migrated to the lower chamber were counted using Trypan blue.

### Cell proliferation, viability, cell cycle, and apoptosis

Human ALL cells cultured in a 24-well plate were provided with stromal OP9 support unless otherwise indicated. For proliferation and viability analysis, treatment was with PTX008 or DMSO for 3 days. Additional cultures were treated with 5 nM vincristine or in combination with PTX008. Cell number and viability were assessed using manual counting of Trypan blue-excluding cells on day 3. For cell cycle analysis with PTX008, vincristine, or a combination of PTX008 with vincristine, 1 × 10^6^ leukemia cells were plated with complete α-MEM media on a confluent layer of mitotically inactivated OP9 cells, or kept in complete medium. At 72 h, cells were incubated with 10 μM BrdU for 4 h. All cells were removed via trypsinization and stained following the manufacturer’s directions (BD FITC BrdU Flow kit, cat #559619, BD Pharmingen, San Jose, CA). Briefly, cells were fixed with BD Cytofix/Cytoperm for 30 min at RT. Cells were washed with Perm/Wash Buffer prior to incubation with Cytoperm Permeabilization Buffer Plus for 10 min on ice. After fixation and permeabilization, cells were treated with 30 μg RNase for 1 h at 37 °C. Cells were then incubated with 1:50 FITC anti-BrdU for 20 min at room temperature. Cells were washed with Perm/Wash buffer and then resuspended with 20 μL 7-AAD prior to addition of FACS buffer. For apoptosis analysis, LAX56 cells grown on mitomycin-C treated OP9 stromal cells or kept in complete medium were treated for 72 h with drugs, then stained for Annexin-V and 7-AAD according to the manufacturer’s instructions (APC Annexin V Apoptosis Detection kit, cat#640930, Biolegend, San Diego, CA). FACS analysis was used to identify early and late apoptotic cells (Annexin-V+, 7-AAD+). Gating in FSC/SSC was on all single cells. Data in Fig. 6a, b were acquired on a BD Accuri C6 cytometer (BD Biosciences, San Jose, CA); those in Fig. 6c and d were acquired on a BD LSR Fortessa X-20 Cell analyzer.

### In vivo treatment of primary ALL with PTX008 and chemotherapy

All animal experiments were carried out in concordance with Institutional IACUC and NIH guidelines. 2 × 10^6^ LAX57 ALL cells were injected i.v. into female NSG mice. On day 7 after transplant, mice were treated intraperitoneally 5× per week with PBS or 5 mg/kg PTX008, or 0.5 mg/kg vincristine once per week, or a combination therapy of daily 5 mg/kg PTX008 and once per week 0.5 mg/kg vincristine. Mice (*n* = 6 mice per group) were treated up to day 35 post-transplant and weighed daily during treatment. When controls lost 10% weight compared to the previous day, all mice were sacrificed. White blood cells from peripheral blood, spleen and bone marrow were analyzed by flow cytometry after red blood cell lysis with BD Pharm Lyse (BD Pharmingen, San Jose, CA). 1 × 10^6^ cells were stained with 1.25 μg CD19 FITC (clone H1B19, Biolegend, San Diego, CA), 0.5 μg LyG FITC (clone 1A8, BD Pharmingen, San Jose, CA), 0.5 μg CD11c PE (clone N418, eBioscience, San Diego, CA), 0.2 μg CD11b (clone M1/70, Biolegend, San Diego, CA), 0.9 μg Galectin-1 (cat 101,566, Genetex, Irvine, CA) conjugated to CF647, or the appropriate isotype controls.

### Statistical analysis

Data shown as mean ± SEM. Statistical analysis was performed using GraphPad Prism 5.0 and 7.0. A *p* value of ≤0.05 was considered significant.

## Results

### Galectin-1 expression in BP-ALL

Based on four ALL cell lines and patient samples, Juszczynski et al. reported that MLL-rearranged ALLs selectively express Galectin-1 [7]. Gene expression profiling by Coustan-Smith et al. [18] confirmed significantly elevated Galectin-1 mRNA in all 23 ALL samples with MLL rearrangement compared to controls. Meta-analysis of that data set however (Fig. 1a) shows that individual samples within other subtypes of ALL also contain high Galectin-1 mRNA levels including non-MLL rearranged samples, compared to normal bone marrow CD19^+^CD10^+^ cells from 4 healthy donors. In agreement with this, Western blotting (Fig. 1b) showed that Galectin-1 protein was present in different non-MLL rearranged BP-ALLs including TXL2, a Ph-positive ALL; diagnosis sample LAX56, relapse sample LAX57, and diagnosis sample US7.

We additionally evaluated TXL2 and US7 for Galectin-1 mRNA levels using real-time PCR. The human BP-ALL cells used in our studies are dependent on stromal support and have a high percentage of dying cells in the absence of a feeder layer when early freeze-downs are used. We and others use the murine bone marrow stromal cell line OP9 to stimulate the growth and also to promote drug resistance development of such human BP-ALL cells (e.g., [15–17, 20–22]). We compared Galectin-1 mRNA in ALL cells kept in complete medium without OP9 cells to those cultured with stromal support, but expression was comparable under these conditions (Fig. 1c). Interestingly, stimulation of US7 BP-ALL cells by the addition of exogenous Galectin-3 induced an increase in Galectin-1 mRNA expression (Fig. 1c, compare US7 control GST to GST-Galectin-3), indicating that in some ALLs, its levels are likely to be regulated by external factors.

Flow cytometric analysis was used to confirm expression of Galectin-1 by various ALL subtypes. Because of a possible low-affinity association of Galectin-1 with the cell surface, we compared the signals of cell surface Galectin-1 with and without chemical cross-linking. As shown in Fig. 1d, DTSSP cross-linking had no measurable effect on Galectin-1 detection, indicating that Galectin-1 is bound with relatively high affinity to these cells. Further FACS analysis confirmed that ALLs other than the MLL subtype express Galectin-1. BM50, from a patient who had relapsed on anti-CD19 CAR-T cell therapy, contained a high percentage of cells with cell surface Galectin-1, whereas ICN06, an ETV6-RUNX positive ALL had the lowest percentage. All the cells in these ALL samples except ICN06 were positive for intracellular Galectin-1 (Fig. 1e).

As increased levels of Galectin-1 were reported in serum samples of lymphoma and osteosarcoma patients [23], we also compared control and ALL bone marrow and peripheral blood plasma samples for Galectin-1. No statistically significant differences were found between ALL and control samples (Fig. 1f, also see Additional file 1: Figure S1). Bone marrow plasma had high levels of Galectin-1 (average, 163.6 ng/ml) compared to blood plasma (average 79.7 ng/ml). We found much higher Galectin-1 levels in normal blood plasma compared to those reported by others in serum (up to 7 ng/ml in healthy people e.g. [24]). This could be caused by depletion of Galectin-1 in serum during coagulation, as it was reported to bind to Factor VIII [25].

### PTX008 inhibition of Galectin-1 binding

To investigate the function of Galectin-1 in ALL cells we made use of a specific small molecule inhibitor of Galectin-1, PTX008 (also known as 0118, OTX008). PTX008 is a non-peptidic, calix (4)arene-based topomimetic of Anginex that acts as an allosteric inhibitor of Galectin-1 by binding to the lectin at a site opposite of the carbohydrate binding domain [12]. PTX008 has been in clinical trials where it was found to be well-tolerated [26] [27].

**Fig. 2:**
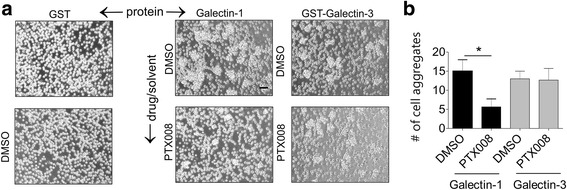
PTX008 inhibits binding of Galectin-1 to the surface of ALL cells. LAX56 cells (2 × 10^4^) were plated without OP9 stroma and treated for 1 h with DMSO or 10 μM PTX008. The ability of human recombinant Galectin-1 or human recombinant GST-Galectin-3 or control GST to cause agglutination of the LAX56 cells was then quantitated by light microscopy. **a** Representative images of cells. Left two panels show controls of cells incubated with GST only or DMSO only. **b** quantification of number of aggregates in each condition. (*p < 0.05)

We used an agglutination assay to confirm the specificity of this compound in inhibiting binding of Galectin-1 to BP-ALL cells. As shown in Fig. 2a, LAX56 cells do not spontaneously aggregate in DMSO or in the presence of control GST but exogenously added Galectin-1 or GST-Galectin-3 promote agglutination. Pretreatment with PXT008 significantly inhibited the ability of Galectin-1 but not of Galectin-3 to agglutinate the cells (Fig. 2b). We also tested if PTX008 could inhibit Galectin-1 and Galectin-3 binding to the cell surface of US7 and LAX56 cells by flow cytometry. As shown in Additional file 1: Table S1, PTX008 inhibited recombinant Galectin-1 but not Galectin-3 binding to cell surface glycoconjugates present on US7 and LAX56 cells.

### PTX008 inhibits integrin-mediated adhesion and migration of ALL cells with cell surface Galectin-1 expression

**Fig. 3:**
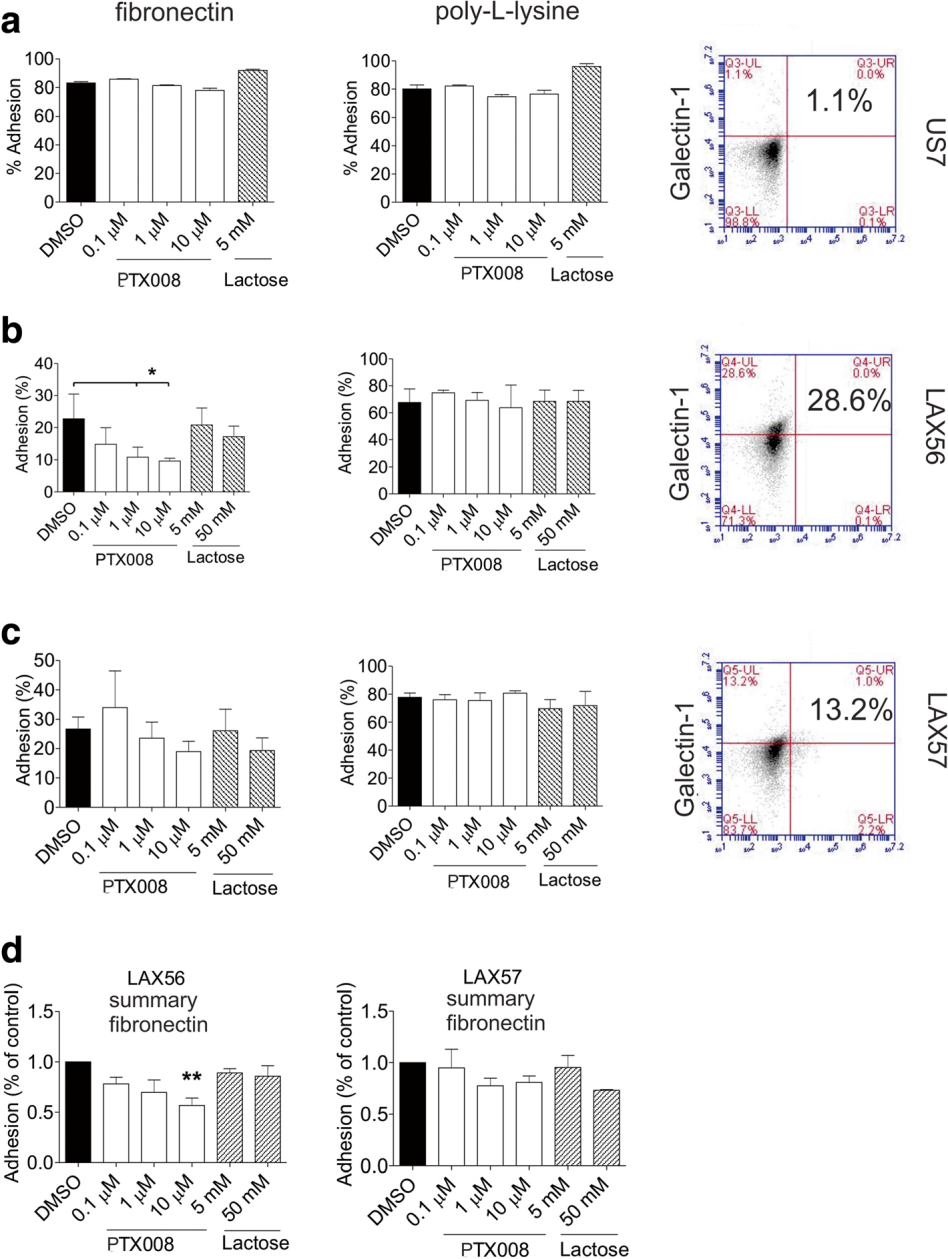
Galectin-1-mediated adhesion to fibronectin is inhibited by PTX008. **a-c** Representative analysis of (**a**) US7, (**b**) LAX56 or (**c**) LAX57 cells adhering to fibronectin (left panels) or poly-L-lysine coated plates (middle panels) in the presence of increasing concentrations of PTX008 or 5 and 50 mM lactose. Error bars: standard deviation. Cell surface staining using FACS (Genetex antibodies) for Galectin-1 on these samples (right panels). Numbers, percentage of cells in the upper left quadrants. *p≤0.05 (95% CI, 1-way ANOVA). **d** Summary fold difference in adhesion of LAX56 and LAX57 to fibronectin and poly-L-lysine coated plates. Percent adhesion was normalized to DMSO control for 3 replicate experiments with 3 or more replicate wells. Error bars, standard error of mean (**p<0.01, 1-way ANOVA)

Extracellular Galectin-1 interacts with glycosylated β1 integrins on BP-ALL Nalm6 cells [28]. As β1 integrins on BP-ALL cells regulate adhesion to extracellular matrix proteins, we also tested if PTX008 would affect integrin-mediated adhesion of BP-ALL cells to fibronectin (FN), a specific integrin α4β1 ligand, or to control poly-L-lysine. We found that PTX008 monotreatment did not affect the viability of the cells under these conditions (also see below). As a positive control for Galectin-1 inhibition, ALL cells were incubated with lactose, a widely used inhibitor for Galectin-1-carbohydrate interactions. As shown in Fig. 3a-c (middle panels), PTX008 had no effect on adhesion of any of the ALLs tested to poly-L-lysine. Also, the drug did not significantly inhibit adhesion of US7 ALL cells (Fig. 3a, left panel), to FN. Treatment of LAX56 cells, which had relatively high cell surface Galectin-1, with PTX008 reduced FN-mediated LAX56 adhesion better than 50 mM lactose (Fig. 3b). Similarly, PTX008 treatment produced a dose-dependent inhibition of LAX57 binding to fibronectin (Fig. 3c). These combined results (Fig. 3d) suggest that PTX008 specifically inhibits the interaction of cell surface Galectin-1 on ALL cells with receptors for fibronectin.

**Fig. 4:**
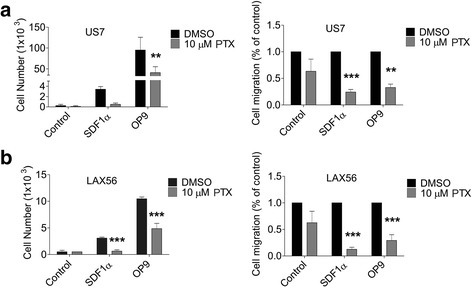
Galectin-1 inhibition reduces ALL cell migration. US7 (**a**) or LAX56 (**b**) cells treated with DMSO or 10 μM PTX008 were allowed to migrate toward αMEM + 2%FBS (control), 200 ng/mL SDF1α, or a confluent layer of irradiated OP9 cells. Left panels, representative experiment. Error bars, standard deviation (**, *p*<0.01, ***, *p*<0.001, 95% CI, 2-way ANOVA). Right panels, summary data from 3 replicate experiments with 3 replicate samples each. Error bars, standard error of mean (**, *p*<0.01, ***, *p*<0.001, 95% CI, 2-way ANOVA)

We also investigated if Galectin-1 inhibition with PTX008 affects ALL cell migration. We tested chemotaxis to SDF1α (CXCL12), as BP-ALL cells exhibit high levels of CXCR4 expression and SDF1α, the CXCR4 ligand, is responsible for ALL cell homing to and engraftment in the bone marrow [29]. As shown in Fig. 4a and b, PTX008-treated US7 and LAX56 cells exhibit significantly reduced migration towards SDF1α, when compared to DMSO controls.

OP9 stromal cells strongly stimulate the migration of BP-ALL cells: when BP-ALL cells are added to a stromal OP9 layer, they migrate to and underneath the stromal layer within a time frame of hours. This is mediated, to a large extent, by SDF1α secreted by the stroma [30]. We therefore also tested the effect of PTX008 in interfering with migration of BP-ALL cells to OP9. Consistent with its inhibition of migration towards SDF1α, as shown in Fig. 4, PTX008 also inhibited migration to OP9 cells compared to vehicle control.

### PTX008 effect on BP-ALL proliferation and viability

**Fig. 5:**
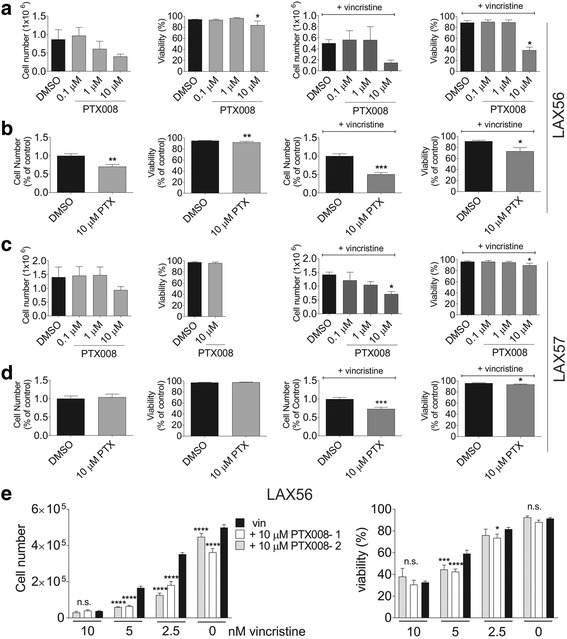
Galectin-1 inhibition combined with vincristine results in increased cytotoxicity to ALL cells in vitro*.* **a** and **c**. Representative analysis of proliferation (left panels, cell numbers) and viability (right panels) of LAX56 (**a**) and LAX57 (**c**) treated for 72 h with PTX008 alone (top panels) or PTX008 plus vincristine (bottom panels) in the presence of OP9 stromal support. Error bars, standard deviation (*p<0.05, 95% CI, 1-way ANOVA). **b** and **d** Summary fold difference LAX56 (**b**) and LAX57 (**d**). Cell number and viability was normalized to DMSO or vincristine only control for 3 replicate experiments with 3 replicate samples. Error bars represent standard error of mean (*, *p*<0.05; **, *p*<0.01; ***, *p*<0.001, two-tailed t-test). **e** Cell counts (left panel) and viability (right panel) of LAX56 in combination treatment for 72 h with a fixed amount of 10 μM PTX008 (− 1 and − 2: drug from different sources) and the indicated concentration of vincristine in the presence of mitotically inactivated (mitomycin C-treated) OP9 stroma. Error bars, SD. One experiment, triplicate wells (*, *p*<0.05; ***, *p*<0.001, ****, *p*<0.0001, compared to vincristine only or DMSO [0 nM vincristine]). Two way ANOVA, Dunnett’s multiple comparison test

We next investigated the effect of Galectin-1 inhibition by PTX008 on ALL cell proliferation and viability. As shown for LAX56 in Fig. 5a-b (left panels) and for LAX57 in Fig. 5c-d (left panels), based on cell numbers, PTX008 was cytostatic for LAX56 (Fig. 5a, b) and for US7 (Additional file 1: Figure S2a). The presence of OP9 stromal cells, as used here, strongly protects BP-ALL cells against eradication when these are treated with chemotherapeutic drugs [15–17]. In agreement with this, in the presence of irradiated OP9 stroma, mono-treatment with this drug at concentrations up to 10 μM had no, or only a small effect on cell viability (Fig. 5a-d).

We also tested a combination treatment with vincristine. LAX56 cells were relatively resistant to a 3-day treatment with 5 nM vincristine (DMSO samples) but a combination with 10 μM PTX008 significantly reduced viability and cell proliferation (Fig. 5a, b right panels). Similarly, the combination also affected LAX57 (Fig. 5c, d right panels) and US7 as well as TXL2 (Additional file 1: Figure S2a, b, bottom panels). Moreover, the inclusion of 10 μM PTX008 allowed a dose reduction of vincristine, with 2.5 nM vincristine combination, achieving a similar cytostatic effect (Fig. 5e, left panel) as monotreatment with 5 nM vincristine.

Nilotinib is a targeted tyrosine kinase inhibitor of BCR-ABL1 that is used to treat Ph-chromosome positive leukemias. We also treated TXL2 cells with the combination of PXT008 and nilotinib. This analysis showed that the cytostatic effect of 20 nM nilotinib was enhanced by the presence of PTX008 (Additional file 1: Figure S2 panel c).

In head and neck SQ20B and A2780-1A9 ovarian cancer cell lines, PTX008 treatment resulted in a decreased level of Galectin-1 expression at 48 and 72 h and reduced phospho-Erk starting as soon as 2 h post-treatment [11]. Because PTX008 interferes with Galectin-1 dimerization [12], and Galectin-1 dimers promote H-Ras nanoclustering in epithelial cells [31–33] we also analyzed Galectin-1, pSrc and pErk1/2 protein levels in lysates of BP-ALL cells treated with PTX008. However, we did not measure marked changes in pSrc or Galectin-1 levels, although pErk1/2 was clearly decreased after 24 h of drug exposure (Additional file 1: Figure S3).

### PTX008 treatment affects BP-ALL cell cycle

**Fig. 6:**
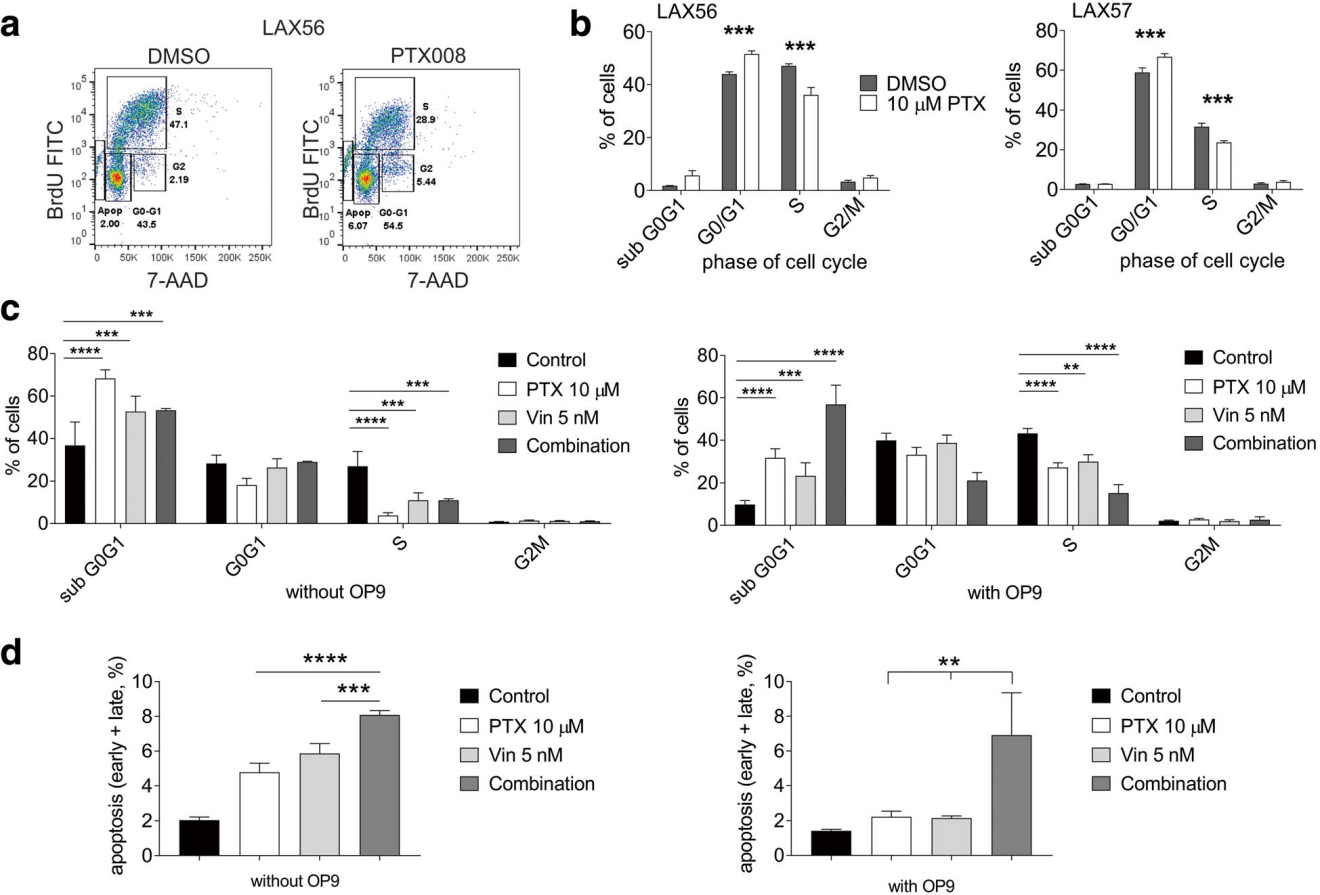
Inhibition of Galectin-1 results in cell cycle arrest. ALL cells grown on (mitotically inactivated, irradiated) OP9 stromal cells were treated with DMSO or 10 μM PTX008, as indicated, for 72 h. **a** Representative flow cytometry images of BrdU and 7-AAD staining of LAX56 cells. Gates represent apoptotic/subG0 (Apop), G0/G1 phase (G0-G1), S phase (S), and G2/M phase (G2) of the cell cycle. **b** Overall cell cycle analysis of LAX56 and LAX57 cells. Graphs are combined data from 3 replicate experiments with 3 replicate samples. Error bars, standard error of mean (*p*<0.001, 95% CI, 2-way ANOVA). **c**, **d**. LAX56 treated for 72 h with 10 μM PTX008, 5 nM vincristine, or a combination of both and control, DMSO treated cells in the absence or presence of (mitotically inactivated, mitomycin-C-treated) OP9 stromal cells as indicated below the panels. Error bars, standard error of mean of triplicate samples. **c** Cell cycle analysis performed as in panel (**a**). (**, *p*<0.01, ***, *p*<0.001, ****, *p* < 0.0001 95% CI, 2-way ANOVA, control versus single or combination treatment). **d** Percentage of apoptotic cells measured by staining for Annexin V and 7-AAD (combination treatment compared to other samples **, *p*<0.01, ***, *p*<0.001, 95% CI, 1-way ANOVA)

We next tested if treatment with PTX008 affects the cell cycle of ALL cells when these were co-cultured with OP9 stromal cells. LAX56 and LAX57 were treated with 10 μM PTX008 for 72 h and then assessed for cell cycle using BrdU and 7-AAD. DMSO-treated control cells showed active DNA synthesis, with a relatively high percentage of cells in S phase. Interestingly, PTX008 treatment significantly reduced this (Fig. 6a, b).

We also compared the effect of PTX008 on BP-ALL cells with and without OP9 stroma. Figure 6c (compare left and right panels, control DMSO-treated samples,) illustrates the impact of the presence of the stromal cells: without OP9 stromal support, more cells with < 2 N DNA content (apoptotic/necrotic- subG0G1) were present. PTX008 treatment had a rather dramatic effect on increasing this population in the absence of OP9 cells but also had a marked effect even in the presence of their support (Fig. 6c, PTX 10 μM samples, compare left and right panels sub G0G1).

Vincristine is a vinca alkaloid that binds to microtubules and spindle proteins in S-phase and interferes with mitosis. As shown in Fig. 6c (vin samples in left and right panels) and as expected, vincristine treatment affected the proliferation of the ALL cells more strongly when no OP9 was present, reducing the percentage of cells in S phase. When we combined the PXT008 and vincristine treatment on cells without stromal support, no additional toxicity was observed (Fig. 6c, combination, left panel). However, because the overall number of healthy cells was higher in the OP9 co-cultures, increased sub G0G1 and decreased S phase cell numbers were measurable in the combination-treated ALL cultures (Fig. 6c, right panel, combination samples).

In addition, we measured apoptosis using FACS and staining for Annexin V and PI for the single and combination drug treatments. Fig. 6d shows that, compared to cells grown on OP9 stroma (right panel), as expected, the percentage cells that were apoptotic in the absence of stromal cells (left panel) was higher. Without stroma, single and combination treatment resulted in increased apoptosis, but the effect of combination treatment was most prominent when the BP-ALL cells were protected by the OP9 cells (Fig. 6d, right panel).

### Addition of PTX008 to vincristine treatment decreases leukemia burden in mice

**Fig. 7:**
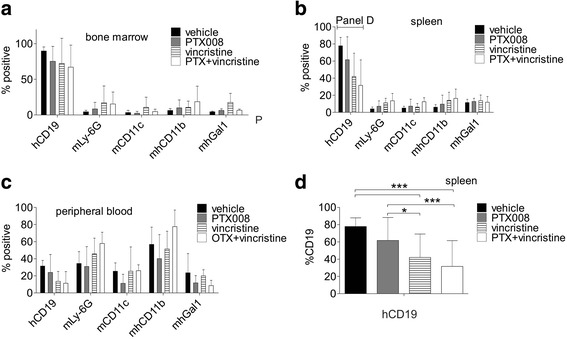
Treatment with PTX008 and vincristine reduces leukemia burden in mice. NSG mice on d7 after transplant with 2 × 10^6^ primary LAX57 ALL cells were treated with vehicle control; 5 mg/kg PTX008 5× per week; 0.5 mg/kg vincristine 1×/ week and 5 mg/kg PTX008 plus 0.5 mg/kg vincristine for 28 days. All mice were euthanized when PBS vehicle control mice exhibited ≥ 10% loss in body weight as compared to the previous day. **a**-**c** Percent of human (h) CD19+ leukemia, murine (m) Ly-6G granulocytes, murine monocyte/dendritic cells, murine/human monocytes, and murine/human Galectin-1 found within (**a**) the bone marrow, (**b**) spleen, (**c**) peripheral blood at the end of the treatment period. **d** Percent of human CD19+ leukemia cells in the spleen of control and treated mice (**, *p*<0.01, ***, *p*<0.001, 95% CI, 2-way ANOVA)

Since PTX008 inhibited the proliferation of ALLs, we next investigated if PTX008 treatment could increase the cytotoxic effect of vincristine in an in vivo NSG transplantation model. We used the diagnosis BP-ALL LAX57 based on its relatively high Galectin-1 expression and sensitivity to vincristine treatment in vitro. After transplant, BP-ALL cells were allowed to proliferate for a one-week period. We then started treatment with PTX008 alone, vincristine alone, or a combination of PTX008 and vincristine for a total of 28 days and monitored body weight of the animals over the treatment period (Additional file 1: Figure S4a and b). At the end of the treatment, mice were euthanized and bone marrow, blood and spleen analyzed for leukemia burden by FACS for human CD19. We also examined expression of CD11c and Ly-6C as markers for murine myeloid cells, and CD11b as leukocyte marker, to address the possibility that inhibition of endogenously produced Galectin-1 activity could affect murine hematopoiesis. As shown in Fig. 7a, c, there was no significant difference between any of the treatments with respect to myeloid markers, or for leukemia burden in blood or bone marrow. This indicates that vincristine monotreatment was not able to control proliferation of these BP-ALL cells in vivo*.* Combination-treated mice did have the lowest spleen weight, which corresponded with a significantly (*p*≤0.001) reduced percentage of CD19+ cells in the spleen compared to PTX008 only treated mice (Fig. 7b, d).

## Discussion

Our data show that subclasses of BP-ALL other than the one characterized by MLL rearrangements express Galectin-1, but that within every subclass, there is a wide range of expression values in the individual samples. The cause of this is unknown, because factors that regulate Galectin-1 mRNA transcription in normal and leukemic B cell precursors have not been identified. In normal mouse B-cell development, high expression levels of Galectin-1 (also known as L14 lectin) mRNA in the pre-B cell stage decrease notably as the cells further mature, and thus high levels may just reflect an earlier precursor B phenotype [34]. The B-lineage transcription factor Pou2AF1 (OCAB, Bob1) regulates Galectin-1, because B-lineage cells lacking OCA-B have reduced Galectin-1, but this is at the level of Galectin-1 cell surface protein expression and not Galectin-1 mRNA [35]. Hypoxia increases Galectin-1 in multiple myeloma [36] but BP-ALL cells in co-culture with OP9 at 22% and 3% O2 contained comparable Galectin-1 protein levels (Additional file 1: Figure S5a). Also, in contrast to Galectin-3 [5], Galectin-1 levels remained unchanged when US7 cells were treated with vincristine, or TXL2 cells were exposed to nilotinib (Additional file 1: Figure S5b). However, stimulation of BP-ALL cells with exogenous GST-Galectin-3 did induce an increase in endogenous Galectin-1 mRNA in US7 cells. We speculate that strong cross-linking of glycoproteins on the cell surface is needed to induce increased Galectin-1 in cells that have low endogenous expression. We previously showed that Galectin-3 made by the OP9 stroma cells is taken up by BP-ALL cells [5], so if the secretion of Galectin-3 in bone marrow stroma is regulated, this in turn could also regulate Galectin-1 mRNA production. If this actually happens in the bone marrow is difficult to determine, as there are many different niches that could secrete Galectin-3: bone marrow stroma consists of multiple cell types including osteoblasts, adipocytes, pericytes, endothelium lining vessels of different types, as well as other hematopoietic elements including tissue-resident macrophages, and osteoclasts [37–39].

We found that BM plasma contains higher levels of Galectin-1 than PB plasma. Mature resident B cells that are present in bone marrow [40] could be one source of Galectin-1 [35]. Bone marrow stromal cells are another source of Galectin-1. We found that TXL2 BP-ALL cells also secrete some Galectin-1 (Additional file 1: Figure S5c,TXL2), and thus BP-ALL cells could contribute to the Galectin-1 found in patient BM plasma. However, the amount of Galectin-1 in control and patient plasma was comparable, indicating that the contribution of the BP-ALL cells is likely to be a minor percentage of the total secreted Galectin-1.

Our studies indicate that Galectin-1 contributes in a number of ways to BP-ALL cell survival. Directed migration and adhesion to bone marrow stromal cells and ECM is known to protect ALL cells against cell death during chemotherapy, and SDF1α is the key chemoattractant and retention factor for leukemic and normal hematopoietic cells in the bone marrow. Thus, ALL survival could be indirectly promoted by bone marrow microenvironment-secreted Galectin-1, which could interact with glycoproteins on the surface of BP-ALL cells and promote chemotaxis: we found that inhibition of Galectin-1 by PTX008 reduced migration towards SDF1α and towards OP9 stroma. This is consistent with the effect of exogenous Galectin-1 on other cell types including U87 glioma cells and umbilical cord blood-derived mesenchymal stem cells, for which it promotes motility [41, 42]. That activity is carbohydrate-dependent, as lactose reversed the increased migration observed with addition of exogenous Galectin-1 [41, 42], which is consistent with our data.

The molecular mechanisms through which PTX008 interferes with adhesion and migration of these cells will need to be further explored. Adhesion of BP-ALL cells to VCAM-1 and FN depends on integrin α4β1 and is known to mediate chemo-protection [43]. We found that PTX008 inhibited integrin α4β1-mediated adhesion to fibronectin. As Rossi et al. [28] reported that α4β1 is a counter-receptor on BP-ALL cells for Galectin-1, this effect of PTX008 appears to be direct.

Although migration of BP-ALL cells to OP9 stroma also depends on integrins including α4β1, it is more difficult to attribute a direct contribution to PTX008 in inhibiting migration to SDF1α, which binds specifically to CXCR4 on BP-ALL cells. CXCR4 carries N-glycosylation on its extracellular domains [44, 45] but no interaction of CXCR4 with Galectin-1 has been reported. We evaluated the effect of PTX008 treatment on cell surface expression of CXCR4 (Additional file 1: Figure S6) but did not find a significant effect on percentage of cells expressing CXCR4 or their MFI for CXCR4, either on OP9 or not. As expected, CXCR4 cell surface MFI levels were higher in cells not co-cultured with OP9 cells, as these produce SDF1α that binds to CXCR4 and causes internalization (Additional file 1: Figure S6 panel b).

Signaling through CXCR4 does increase integrin α4β1 affinity via an inside-out mechanism [46], but this cross-talk would not explain why binding of Galectin-1 to the integrin β1 chain affects CXCR4 signaling. Thus, other poly/multiantennary LacNAc- containing cell surface proteins that can bind Galectin-1 and which were identified on Nalm6 BP-ALL cells [47] could affect CXCR4 signaling. For example, in the T-cell line Jurkat, CXCR4 and the hematopoietic-specific tyrosine phosphatase CD45 can be co-immunoprecipitated. Reducing levels of CD45 or inhibition of lipid raft formation and CD45/CXCR4 association through methyl-β-cyclodextrin treatment decreased chemotaxis to SDF1α [48]. Thus, as treatment with extracellular Galectin-1 causes polarized clustering of CD45 on dendritic cells [49], one possible mechanism through which PTX008 can inhibit migration to SDF1α is if reduces the binding of Galectin-1 to CD45, leading to reduced polarization of CXCR4, which is needed for efficient migration of HSPC [50]. A distinct mechanism could involve inhibition of intracellular Galectin-1 functions by PTX008. This has been reported in A498 and breast cancer cell lines, as well as in normal lymphatic endothelial cells, where endogenous Galectin-1 regulates migration through different mechanisms [51–53].

PTX008 treatment at 10 μM was cytostatic for ALL cells. This result agrees with studies in other cell types [11, 13, 54]. For example, PTX008 treatment of human umbilical vein-derived endothelial cells resulted in a significant reduction in cell proliferation [54, 55]. Also, growth of human ovarian MA148 and mouse melanoma B16 tumors were significantly reduced with treatment with PTX008 in vivo [55]. Similarly, PTX008-treated A27080-1A9 ovarian cancer xenografts showed reduced cell proliferation at comparable levels to that of xenografts treated with cisplatin and docetaxel alone [11].

PTX008 mono-treatment affected the cell cycle of BP-ALL cells and reduced the percentage of cells synthesizing DNA. Previous work by Astorgues-Xerr et al. showed that head and neck SQ20B and ovarian A2780-1A9 cancer cells accumulated in the G2/M phase of the cell cycle after a similar treatment [11]. Thus, although our studies agree that one of the activities of PTX008 treatment is to inhibit cell cycle, the phase at which this happens may depend on the cell type (i.e., differences between leukemias and carcinomas) or correlate with for example the presence of different oncogenic mutations. In this context, it is also important to note that Galectin-1 is found at multiple intracellular locations including the nucleus, where it is involved in RNA splicing [56], and in the cytoplasm [57]. Mechanistically, it is therefore possible that intracellular inhibition of Galectin-1 interactions has more than one effect. We found that the inclusion of 10 μM PTX008 modestly enhanced the cytotoxicity of vincristine, with the combination treatment increasing both the percentage of apoptotic/necrotic cells as well as reduction of the percentage of cells in S phase. Although vincristine inhibits the S phase of cell cycle by interfering with microtubule polymerization essential for spindle pole formation, it also promotes lysosomal cell death (LCD) [58]. Galectin-1 punctate structures are known to form at the lysosome during the early stages of LCD [59], and if this is protective to a cell, its inhibition by PTX008 could facilitate cell death through this mechanism.

In an NGS mouse transplant model, the combination of PTX008 with vincristine reduced leukemia cell numbers in the spleen. Thus, these experiments provide a proof-of-principle that targeting Galectin-1 in ALL could be useful therapeutically, because this interferes with protection from bone marrow stromal cells, affects the ALL cell cycle, and possibly also affects immune cell function as was reported for a lymphoma model [8]. However, we needed relatively high concentrations of PTX008, in the micromolar range, to obtain a biological effect even in vitro in tissue culture. This may relate to pharmacokinetic properties of the drug, the high concentrations of Galectin-1 secreted, or to the fact that lectins are difficult to target therapeutically because carbohydrate-lectin interactions are weak. Additionally, attenuated responses to PTX008 in vivo may be a result of Galectin-3 compensation for Galectin-1 inhibition, because protective bone marrow stromal cells secrete both lectins.

Our previous Galectin-3 data, in conjunction with the currently reported effects of Galectin-1 on BP-ALL cell proliferation, migration, and chemo-protection suggest that the Galectin-1 and Galectin-3 endogenously generated in ALL cells have important contributions to their survival and are drugable targets. Derivatives of PTX008 with improved IC50 values were reported that inhibited MA148 ovarian cancer cell line proliferation 10-fold more potently than PTX008 [60]. Moreover, PET imaging in mice showed accumulation of two of these derivatives in bone [60]. In the future, it may be therefore feasible to test out a strategy of combined inhibition of Galectin-1 and Galectin-3 function using small molecule inhibitors or monoclonal antibodies [61–63].

## Additional file

Additional file 1: **Figure S1.** ELISA for measurement of Galectin-1 in plasma samples. **Table S1.** PTX008 inhibits recombinant Galectin-1 but not Galectin-3 binding to cell surface glycoconjugates present on US7 and LAX56 cells. **Figure S2.** Galectin-1 inhibition is cytostatic and cytotoxic to Ph-negative US7 and Ph-positive TXL2 ALL cells. **Figure S3.** PTX008 treatment decreases pErk in BP-ALL cells. **Figure S4.** NSG mice transplanted with BP-ALL treated with PTX008 and vincristine. **Figure S5.** Galectin-1 expression in BP-ALL cells exposed to drugs or hypoxia. **Figure S6.** Effect of PTX008 on CXCR4 cell surface expression. (DOCX 12500 kb)

## Abbreviations

BP-ALL: B-cell precursor acute lymphoblastic leukemia
FN: Fibronectin
LCD: Lysosomal cell death
MFI: Mean fluorescent intensity
Ph: Philadelphia
RT: Room temperature
