# Supplementary material for: Treatment of B-cell precursor acute lymphoblastic leukemia with the Galectin-1 inhibitor PTX008
Source: J Exp Clin Cancer Res. 2018 Mar 27;37:67. doi: 10.1186/s13046-018-0721-7 (PMC5870532; doi:10.1186/s13046-018-0721-7)
Supplement: Supplementary file 1 — Figure S1. ELISA for measurement of Galectin-1 in plasma samples. Table S1. PTX008 inhibits recombinant Galectin-1 but not Galectin-3 binding to cell surface glycoconjugates present on US7 and LAX56 cells. Figure S2. Galectin-1 inhibition is cytostatic and cytotoxic to Ph-negative US7 and Ph-positive TXL2 ALL cells. Figure S3. PTX008 treatment decreases pErk in BP-ALL cells. Figure S4. NSG mice transplanted with BP-ALL treated with PTX008 and vincristine. Figure S5. Galectin-1 expression in BP-ALL cells exposed to drugs or hypoxia. Figure S6. Effect of PTX008 on CXCR4 cell surface expression. (DOCX 12500 kb) [file 13046_2018_721_MOESM1_ESM.docx]

**Additional File 1**


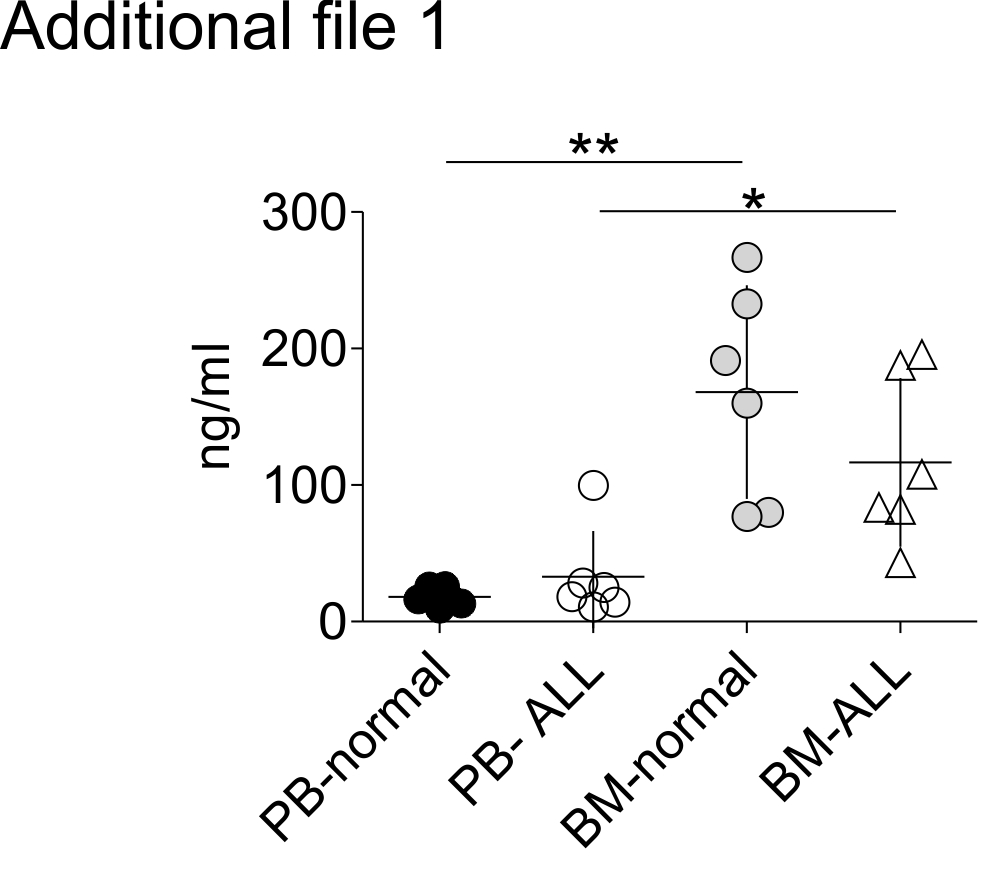


**Figure S1.** ELISA for measurement of Galectin-1 in plasma samples. Galectin-1 measurements on the same samples as in Figure 1f using an ELISA (R&D Systems) for human Galectin-1.

| Cells | inhibitor | recombinant protein | detection antibody | MFI |
| --- | --- | --- | --- | --- |
| US7 |  |  | IgG | 545 |
|  |  |  | Gal1 | 1427 |
|  | lactose |  | Gal1 | 1438 |
|  | PTX008 |  | Gal1 | 1387 |
|  | DMSO | rhGal1 | Gal1 | 2052 |
|  | PTX008 | rhGal1 | Gal1 | 1565 |
|  | lactose | rhGal1 | Gal1 | 1830 |
|  |  |  | IgG | 570 |
|  | DMSO |  | Gal3 | 1246 |
|  | PTX008 |  | Gal3 | 1487 |
|  | DMSO | rhGal3 | Gal3 | 7056 |
|  | PTX008 | rhGal3 | Gal3 | 8740 |
|  |  |  |  |  |
| LAX56 |  |  | IgG | 564 |
|  |  |  | Gal1 | 2819 |
|  | lactose |  | Gal1 | 2764 |
|  | PTX008 |  | Gal1 | 2456 |
|  | DMSO | rhGal1 | Gal1 | 3610 |
|  | PTX008 | rhGal1 | Gal1 | 2979 |
|  | lactose | rhGal1 | Gal1 | 2611 |
|  |  |  | IgG | 570 |
|  | DMSO |  | Gal3 | 1291 |
|  | PTX008 |  | Gal3 | 1347 |
|  | DMSO | rhGal3 | Gal3 | 5944 |
|  | PTX008 | rhGal3 | Gal3 | 5747 |

**Table 1**. PTX008 inhibits recombinant Galectin-1 but not Galectin-3 binding to cell surface glycoconjugates present on US7 and LAX56 cells. DMSO or 10 μM PTX008 was added to the indicated leukemia cells. After 1 hour, 50 mM lactose and 20 μM recombinant protein was added. Cells were incubated for 24 hours, after which flow cytometry using the indicated antibodies was performed. MFI, mean fluorescent intensity.


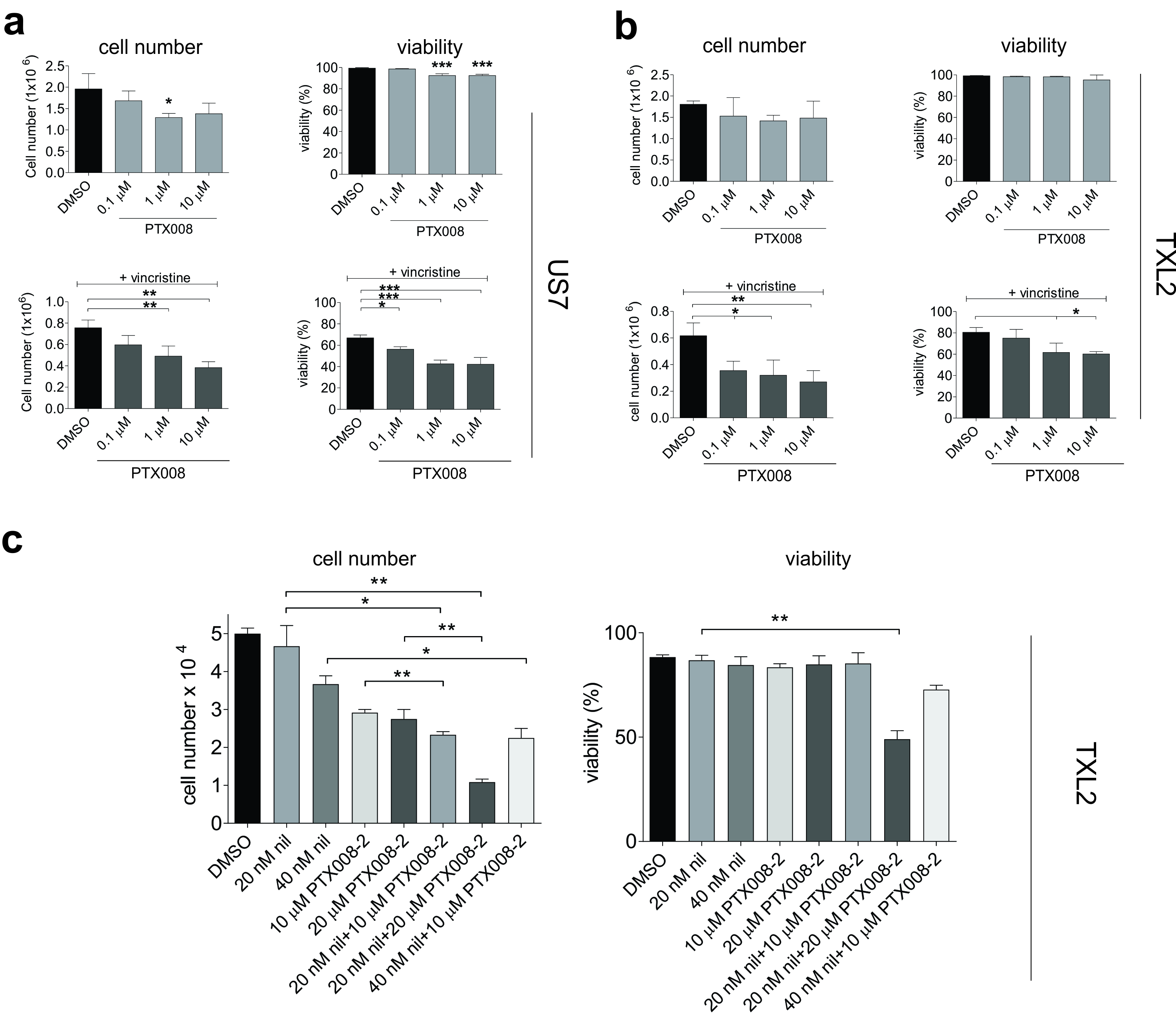


**Figure S2.** Galectin-1 inhibition is cytostatic and cytotoxic to Ph-negative US7 and Ph-positive TXL2 ALL cells. (**a**-**b**) Comparative analysis of proliferation (left panels, cell numbers) and viability (right panels) of (**a**) US7 or (**b**) TXL2 ALL cells treated for 72 hours with PTX008 alone (top panels) or PTX008 plus vincristine (bottom panels) in the presence of OP9 stromal cells. Error bars: standard deviation (*p<0.05; ** p<0.01; ***p<0.001, 95% CI, 1-way ANOVA). (**c**) TXL2 BP-ALL cells co-cultured with mitomycin C-treated OP9 cells were treated for 72 hours with PTX008 alone, with nilotinib alone, or with a combination of the two drugs. Counts include both BP-ALL cells in suspension and associated with the stroma. Single experiment, triplicate wells. Error bars: mean ±SEM. *p<0.05; **p<0.01 for the indicated comparisons.


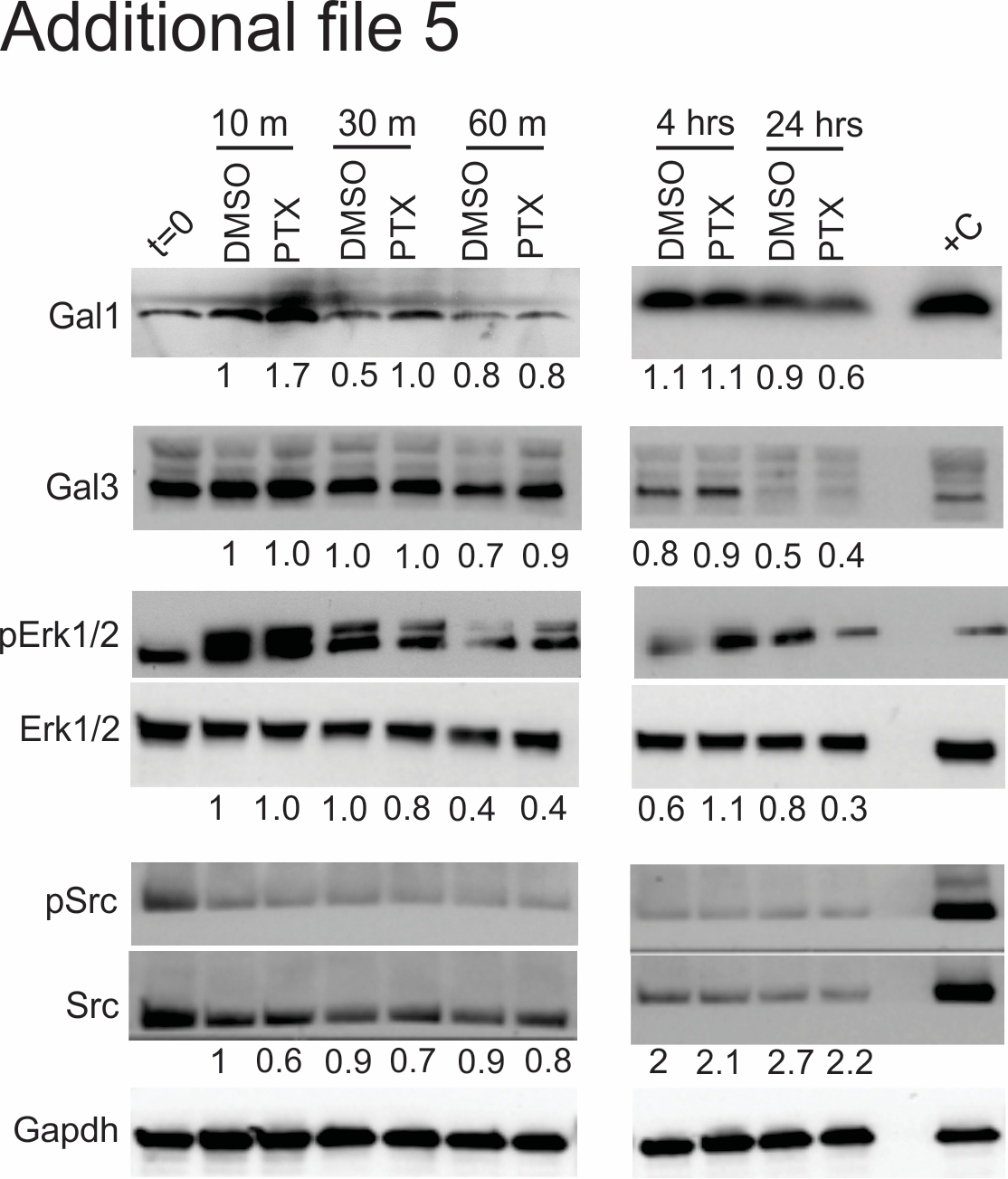


**Figure S3.** PTX008 treatment decreases pErk in BP-ALL cells**.** Serum-starved LAX56 cells were plated on t=0 on irradiated OP9 cells, while treated for the indicated periods of time with DMSO or 10 μM PTX008. Antibodies used are indicated to the left; Gapdh, loading control. Numbers below panels; optical density ratio of scans of WB for Galectin-1 (Gal1) and Galectin-3 (Gal3) to Gapdh and for pErk1/2 to Erk, or pSrc to total Src determined using Image J and normalized to the t=10 minutes DMSO time point. +C = TXL2 cell lysate.

**
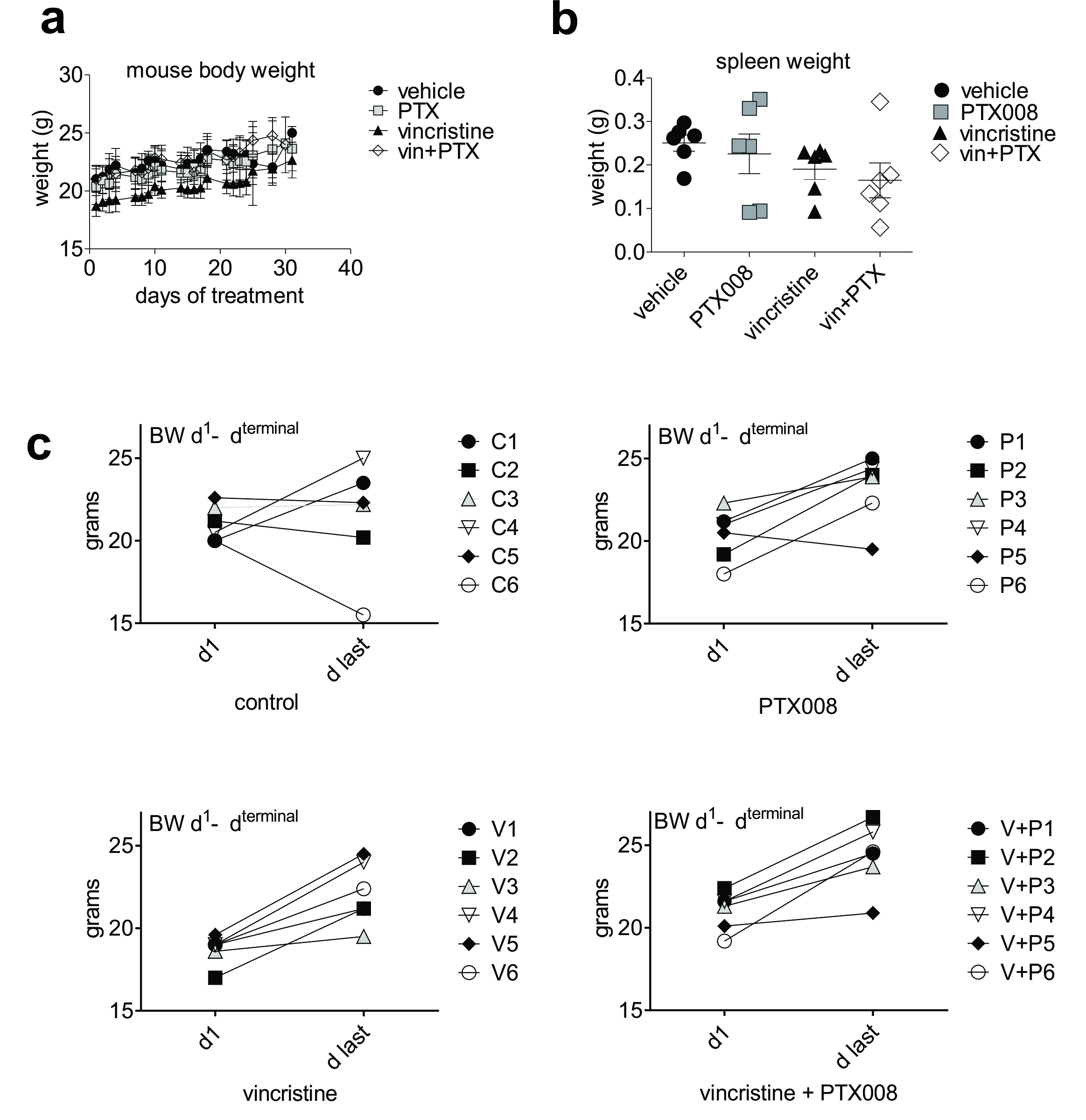
**

**Figure S4.** NSG mice transplanted with BP-ALL treated with PTX008 and vincristine. NSG mice were transplanted with LAX57 (Fig. 7) (**a**) Average body weight of mice over the course of the treatment period with vehicle control; 5 mg/kg PTX008; 0.5 mg/kg vincristine; and 5 mg/kg PTX008 plus 0.5 mg/kg vincristine. (**b**) Average spleen weight, with each individual symbol representing each mouse in the 4 treatment groups. n=6 per group. (**c**) Begin (after first treatment) and end weight (before euthanasia) of individual mice.


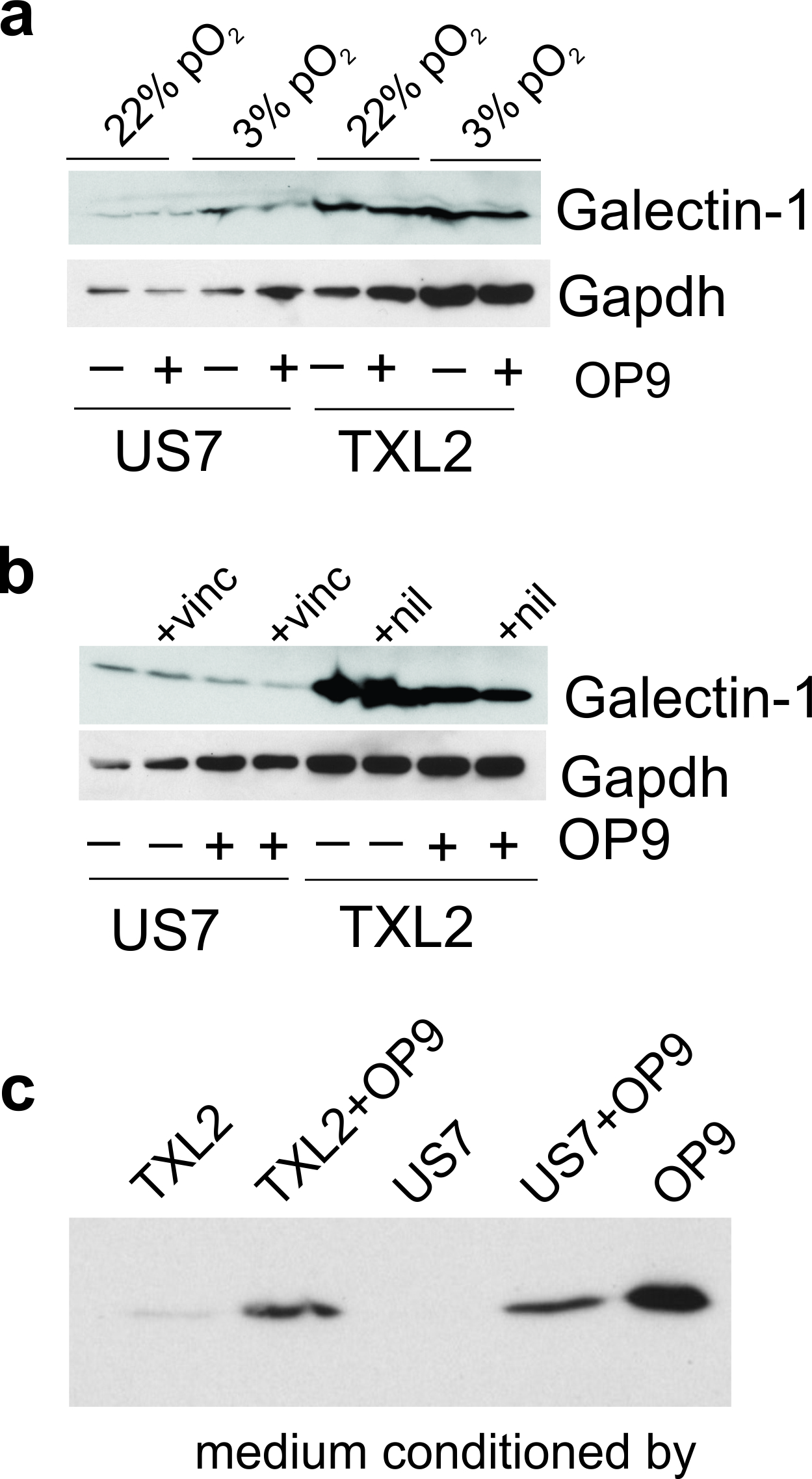


**Figure S5.** Expression of Galectin-1 under different conditions. (**a**) Western blot of human BP-ALL US7 or TXL2 cells under standard growth conditions, for at least 24 hours at ambient pO_2_ (22%) or reduced pO_2_ as indicated, with or without the presence of irradiated OP9 stromal cells. (**b**) Western blot of BP-ALL cell lysates with or without irradiated OP9 stromal cells as indicated. Cells were exposed for 24 hours to 5 nM vincristine, 1 μM nilotinib, or DMSO. Gapdh, loading control. (**c**) The indicated cells or cell combinations (3x10^6^ cells) in a 6-well format were used to condition α-MEM medium without FBS for 24 hours. Cell-free medium was collected and concentrated 50x. 4 μL of the conditioned medium was loaded per well. The Western blot was probed with Galectin-1 antibodies.

**
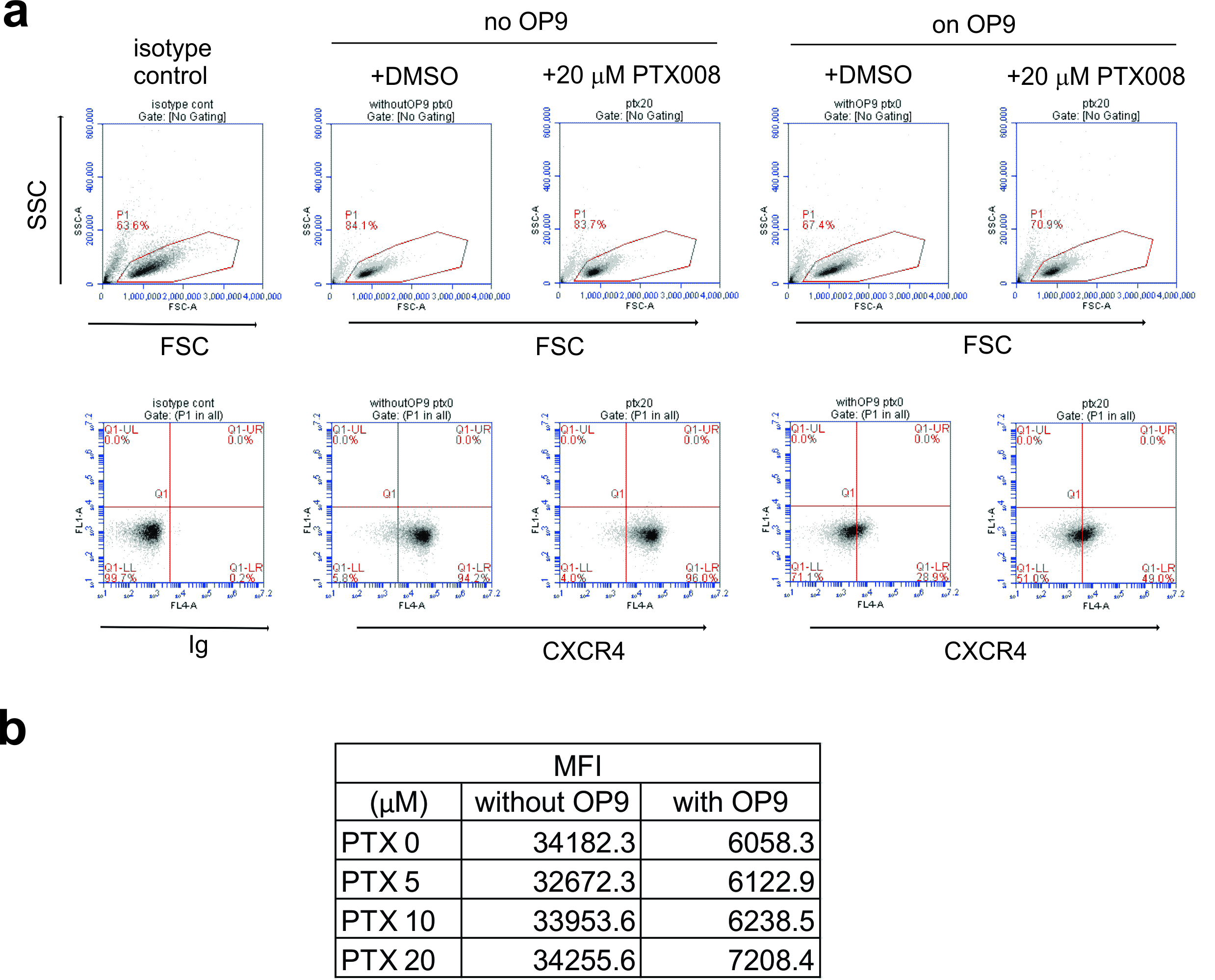
**

**Figure S6.** Effect of PTX008 on CXCR4 cell surface expression. LAX56 cells were plated on mitotically inactivated OP9 cells, or not, as indicated, in α-MEM +2% FBS. Cells were treated with PTX008-2 or with DMSO for 24 hrs. Flow cytometry was with anti-CXCR4 antibodies (anti-CD184; Biolegend cat #306510). (**a**) Representative FACS plots of cells treated as indicated. (**b**) MFI (mean fluorescent intensity) for CXCR4 cell surface staining of cells treated as indicated.
